# The Effects of Aqueous Extract from* Nardostachys chinensis* Batalin on Blood Pressure and Cardiac Hypertrophy in Two-Kidney One-Clip Hypertensive Rats

**DOI:** 10.1155/2017/4031950

**Published:** 2017-10-11

**Authors:** Rayile Aisa, Zhaoxia Yu, Xiangyang Zhang, Dilinuer Maimaitiyiming, Lipeng Huang, Ayshamgul Hasim, Tao Jiang, Mingjun Duan

**Affiliations:** ^1^Cardiac Center, First Affiliated Hospital of Xinjiang Medical University, Urumqi 830054, China; ^2^Department of Internal Medicine, Kuqa County Hospital of Traditional Uyghur Medicine, Kuqa 842000, China; ^3^Department of Pathology, Xinjiang Medical University, Urumqi 830011, China; ^4^Institute of Clinical Medicine Research, First Affiliated Hospital of Xinjiang Medical University, Urumqi 830054, China

## Abstract

**Aims:**

The aim of this study was to investigate the effects of the aqueous extract of* Nardostachys chinensis* Batalin (NCBAE) on blood pressure and cardiac hypertrophy using two-kidney one-clip (2K1C) hypertensive rats.

**Methods:**

2K1C rat models were set up by clipping the left renal artery. Sham-operated rats underwent the same surgical procedure except for renal arterial clipping. 2K1C hypertensive rats were orally given NCBAE at doses of 210, 420, and 630 mg·kg^−1^·d^−1^ for 6 weeks. Twelve weeks after surgery, rat SBP and echocardiographic parameters were measured, cardiac histopathology was assessed, serum NO and LDH were detected, and the expression of Bcl-2 and caspase-3 of left ventricular tissue was assessed by western blot.

**Results:**

Treatment with NCBAE resulted in a decrease of SBP, LVPWd, LVPWs, IVSd, IVSs, LVW/BW ratio, and cardiomyocyte CSA, an increase of LVEF, and inhibition of 2K1C-induced reduction in serum NO and elevation of LDH compared with 2K1C group. NCBAE intervention also showed a significant increase of Bcl-2 expression and reduction of cleaved caspase-3 level dose-dependently in left ventricular tissue.

**Conclusion:**

Our data demonstrate that NCBAE has an antihypertensive property and protective effect on 2K1C-induced cardiac hypertrophy especially at the dose of 630 mg·kg^−1^·d^−1^.

## 1. Introduction

Hypertension leads to many types of organ damage, including cardiac hypertrophy, which increases the risk of heart failure [1, 2], and is associated with increased morbidity and mortality caused by cardiovascular disorders [3]. So, we can see that the hypertensive cardiac disorder is one of the major healthcare problems nowadays. Although there are antihypertensive drugs, such as diuretics, *β*-blockers, calcium channel blockers, and angiotensin converting enzyme inhibitors, which are helpful in controlling blood pressure (BP), they have their potential side effects in application. Natural medicinal plants could be an ideal and safe source to develop effective agents for hypertension and hypertensive cardiac disorders.

*Nardostachys chinensis* Batalin (NCB, same as “Gansong” in Traditional Chinese Medicine and “Sumbul Chine” in Traditional Uyghur Medicine), which belongs to the genus* Nardostachys* (Valerianaceae), is a well-known traditional herbal medicine, enriched with a series of aristolane, nardosinane, guaiane-type sesquiterpenes [4]. The roots and rhizomes of NCB have long been used for their sedative [5], aromatic [5], stomachic [6, 7], and neuron protective effects [8–10]. NCB plays efficient roles in strengthening tendons and nourishing brain and heart, in antinociception, and in neurotrophy as a Traditional Uyghur Medicine [8] and is used for chest tightness, shortness of breath, hypertension, and neurasthenia [6, 11]. Moreover, NCB was clinically used for premature ventricular contractions [12] and showed a good application prospect on arrhythmia [13, 14]. Recently, a study showed that compounds nardosinanone F, nardosinanone I, nardosinonediol, kanshone E, narchinol A, and narchinol B from NCB had protective effects on injured cardiomyocytes of neonatal rats [15]. It also has been reported that NCB has an inhibitory effect on angiotensin II- (Ang II-) induced cardiac hypertrophy [16]. Its cardioprotective efficacy has gradually become the object of attention.

In the progression of hypertensive cardiac disorders, the disappearance of cells by apoptosis is an indispensable process in addition to the proliferation and hypertrophy of cardiac cells [17, 18]. The classic apoptotic pathways include, but are not limited to, intrinsic mitochondrial pathway and extrinsic Fas/FasL death receptor pathway, and they eventually result in apoptosis by activation of the caspases family. Apoptosis is modulated by proapoptotic and antiapoptotic factors. For example, Bcl-2 protein has an antiapoptotic effect, while the activation of caspase-3 results in apoptosis [19]. In addition, nitric oxide (NO) is an endothelium-derived relaxing factor, which has reduced production and decreased bioavailability in hypertension [20]. During the development of hypertension, there are interactions among NO, Ang II, and oxygen free radical. Ang II promotes the production of superoxide anion via increased expression of NADH oxidase and NADPH activation. The vascular contractive effect of Ang II is increased when there is a lack of NO and is weakened during antioxidant therapy. In experimental models of two-kidney one-clip (2K1C) renovascular hypertension, the increased production of Ang II caused by clipped kidneys may scavenge endothelial NO, resulting in the pathophysiological changes and the elevation of BP, vasospasm [21], and left ventricular dysfunction [22]. These 2K1C experimental models are suitable for the evaluation research of cardioprotective drugs [23].

In order to research the possibility of NCB on BP regulation and cardiac protection in 2K1C hypertensive rats, we examined the effects of the aqueous extract of* Nardostachys chinensis* Batalin (NCBAE) on systolic blood pressure (SBP), echocardiographic parameters, cardiomyocyte cross-sectional area (CSA), serum NO levels, and activity of serum lactate dehydrogenase (LDH) and assessed the involvement of cardiac expression of Bcl-2 and caspase-3 in its therapeutic effects in this study.

## 2. Materials and Methods

### 2.1. Herbal Extract and Chemicals

*Plant Material*. The roots and rhizomes of NCB were purchased from Xinjiang Bencaotang Chinese Herbal Pieces Co. Limited (Urumqi, China; batch number: 14060101) and authenticated by Dr. Haiyan Xu, Institute of Traditional Chinese Medicine, Xinjiang Medical University, Urumqi, China. A voucher specimen (XJMUCL-VSN-2014-001) of NCB was deposited in the herbarium of the Central Laboratory of Xinjiang Medical University (Urumqi, China).

*Extract*. The air-dried medical material was crushed to a fine powder (60 mesh). The powder was dissolved in hot distilled water (100°C) and extracted twice with distilled water (tenfold volume) for 1 h at each time, and the decoctions were combined. The solution was filtered and evaporated to yield a brown extract. The yield of NCBAE was 13.90% (w/w). The resulting powder stored at 4°C was diluted according to the doses (calculated and expressed as mg·kg^−1^ body weight: 210, 420, and 630 mg·kg^−1^·d^−1^) needed with distilled water and filtered before use. 

*Chemicals*. Atorvastatin (Ator), which was obtained from Pfizer Inc. (Dalian, China; batch number: J81037), was dissolved in distilled water as a positive control. The dose of Ator for administration was calculated and expressed as mg·kg^−1^ body weight (16.5 mg·kg^−1^·d^−1^). Rabbit anti-rat monoclonal antibody of anti-Bcl-2 was obtained from Abcam® Inc. (Cambridge, England, ab136285). Polyclonal antibody of anti-caspase-3 was obtained from Cell Signaling Technology® Inc. (Danvers, USA, #9662). Polyclonal antibody of anti-*β*-actin was obtained from Bioworld Technology Inc. (Minnesota, USA, AP0060).

### 2.2. Analytical Methods for Chemical Composition of NCBAE

*High-Performance Liquid Chromatography (HPLC) Analysis*. The standard compounds linarin (purity 98.94%) and *β*-sitosterol (purity 98%) were obtained from Chengdu PureChem-Standard Co., Ltd. (Chengdu, China). HPLC-grade reagents, acetonitrile, and methanol were obtained from Sigma-Aldrich (St. Louis, MO, USA). Analytical column used for separation was C_18_ (5 *μ*m, 4.6 mm × 250 mm) from Dalian Elite Analytical Instruments Co., Ltd. (Dalian, China). All other chemicals used in HPLC analysis were of reagent grade.

Determination of linarin and *β*-sitosterol was performed by LC-20AT high-performance liquid chromatography (Shimadzu Corporation, Japan) in the Central Laboratory of Xinjiang Medical University. In brief, a total of 10 mg of NCBAE and 5 ml of methanol were mixed and sonicated for 20 min (100 Hz). After the dissolution, add methanol to 10 ml, shake well, and filter through a 0.22 *μ*m membrane for HPLC analysis. The standard solutions of linarin and *β*-sitosterol were prepared in methanol at a concentration of 0.045 mg/ml and 0.98 mg/ml. Gradient elution was performed with solution A (acetonitrile) and solution B (water) in the following gradient elution program: 0–10 min, 15% of solution A; 11–60 min, 95% of solution A; 61–70 min, 15% of solution A. The dried extract was dissolved in HPLC-grade methanol (1 mg/ml) and filtered through a sterile 0.22 *μ*m syringe filter and 10 *μ*L volume was injected to the HPLC. Flow rate was maintained at 1 ml/min and temperature was adjusted to 25°C.

*Mass Spectrometric Analyses.* Mass spectrometric analyses were performed using an Acquity UPLC (Waters Co., Milford, MA, USA) coupled with a Micromass Quattro Ultima triple quadrupole mass spectrometer (Waters Co., Milford, MA, USA) equipped with an electrospray ion source (ESI) operating in positive mode. Data were acquired with Empower 3 software. Analytical column used for separation was UPLC BEH C_18_ (1.7 *μ*m, 2.1 × 100 mm) from Waters Co. The chromatographic run was performed at 200 *μ*L min^−1^ with a gradient elution of a mobile phase which consisted of solution A (water) and solution B (acetonitrile). Briefly, the experimental system was operated with an ESI interface in positive ionization mode. The cone and desolvation gas flow rates were set at 50 L h^−1^ and 500 L h^−1^, respectively. Capillary voltage was set at 2.98 kilovolts, source temperature was 120°C, and desolvation temperature was 300°C. The cone voltage of linarin and *β*-sitosterol was set at 40 and 50 volts, respectively.

### 2.3. Animals and Treatments

Normotensive male Wistar rats with a body weight ranging between 250 and 300 g were obtained from the Laboratory Animal Center of Xinjiang Medical University. The rats were kept at constant physical ambient conditions (20~25°C, 45% humidity, and regular day : night cycle) and supplied with tap water and standard diet ad libitum.

2K1C renovascular hypertension was induced as described previously [24–26]. Briefly, the rats were anesthetized with chloral hydrate (300 mg/kg, resp., i.p.). A silver clip (0.25 mm ID) was placed around the left renal artery through a midline incision in the model group, resulting in partial occlusion of renal perfusion. Other rats underwent the same surgical procedure except for renal arterial clipping. Systolic blood pressure was measured weekly by Tail-Cuff Blood Pressure System with rats under consciousness. For 2K1C operated Wistar rats, only those whose systolic blood pressure rose equal to and higher than 150 mmHg within 6 weeks were considered to be 2K1C hypertensive rats and enrolled in this experiment [27]. This protocol was reviewed and approved by the Ethical Committee of the First Affiliated Hospital of Xinjiang Medical University.

Treatments were started 6 weeks after surgery and maintained for 6 weeks. Animals were randomly assigned to one of six groups: NCBAE was intragastrically administered at doses of 210 mg·kg^−1^·d^−1^ (NCBAE (L)), 420 mg·kg^−1^·d^−1^ (NCBAE (M)), and 630 mg·kg^−1^·d^−1^ (NCBAE (H)) in three 2K1C groups each, Ator was intragastrically administered at a dose of 16.5 mg·kg^−1^·d^−1^ in the 2K1C group, and distilled water was administered in 2K1C and sham animals for about 6 weeks. At the end of the experiment, all animals were deeply anesthetized with chloral hydrate and blood was drawn by abdominal aortic puncture. The blood samples were centrifuged at 3000 rpm at 25°C for 10 min, and serum was obtained and stored at −80°C for biochemical assays. The hearts were excised, washed with ice-cold saline, and blotted with filter paper and the left ventricles were divided into two halves. One half was fixed in 4% formaldehyde solution for histopathological study, and the other half was stored at −80°C for western blot analysis.

### 2.4. Blood Pressure Measurement

SBP of all the animals was measured before the surgery and once a week after clipping by BP-6 Tail-Cuff Blood Pressure Measuring System (Chengdu Technology & Market Corp., Ltd., Chengdu, China) with rats under consciousness. All animals were kept calm and warmed for 15 min till pulsatory signals were displayed steadily before the SBP measurement. The mean of three measurements was obtained from each rat once a week until the end of the experiment.

### 2.5. Measurements of Echocardiograph

After six-week treatments, rats were weighed, and transthoracic echocardiography was performed in all rats in this study under anesthesia (chloral hydrate, 300 mg/kg, resp., i.p.) with spontaneous respiration. The chest was shaved, and the animal was situated in the supine position. Left ventricle (LV) function was evaluated by echocardiography using Philips HD IIXE (Philips, Netherlands) with a 7.5 MHz probe (S12-4). Two-dimensionally (2D) guided M-mode echocardiographic images in the left ventricular long-axis level were taken. Measurements were averaged from five consecutive beats. The rats of all groups underwent echocardiography after six weeks of treatment. The following parameters were measured: left ventricular posterior wall at end diastole (LVPWd), left ventricular posterior wall at end systole (LVPWs), interventricular septal diameter at end diastole (IVSd), interventricular septal diameter at end systole (IVSs), left ventricular internal diameter at end diastole (LVIDd), left ventricular internal diameter at end systole (LVIDs), percentage of left ventricular fractional shortening (LVFS), and left ventricular ejection fraction (LVEF). Echocardiographic examination was performed by a professional echocardiographer blinded to the study groups.

### 2.6. Organ Weights

After imaging was complete, hearts of rats in each group were removed immediately under anesthesia. Left ventricles were collected and weighed. The organ mass (mg) was normalized by the body weight (g) giving a left ventricular weight/body weight (LVW/BW) ratio index. The index was adopted to prevent variations among different animal sizes.

### 2.7. Histopathological Observations of Heart

The rats' hearts were harvested and fixed in 4% formaldehyde solution for at least 24 h and embedded in paraffin. The paraffin-embedded specimens were cut into sections (3 *μ*m thick) and stained with hematoxylin-eosin. The images were digitally captured (magnification: ×400) using a Leica DM3000 microscope (Leica Biosystems, Germany). Cardiomyocyte stained cross-sectional areas of each group were measured and digitalized by Image-Pro Plus 6.0 software (Media Cybernetics, Silver Spring, MD, USA). CSA/control ratios of each group were calculated and analyzed [28].

### 2.8. Measurement of Serum NO and LDH

Serum NO level was estimated by the nitrate reductase method, and the activity of serum LDH was determined by enzymatic kits. All experimental procedures were strictly in accordance with the manufacturer's instructions (Jiancheng Bioengineering Company, Nanjing, China; NO assay kit: A012, LDH activity assay kit: A020-2).

### 2.9. Measurement of Cardiac Bcl-2 and Caspase-3 Level by Western Blotting

To analyze protein expression, left ventricular tissues were prepared and proteins extracted for western blotting analyses. The protein extracts were boiled in 4 × loading buffer at 100°C for 10 min. Protein extracts of each group were subjected to SDS-PAGE using a 12% polyacrylamide gel, and 40 *μ*g of protein was loaded in each well. The proteins were then transferred onto PVDF membranes and blocked with blocking buffer (Biosharp, Hefei, China; BL535A) for 60 min. The membranes were incubated overnight at 4°C with the rabbit anti-rat monoclonal antibodies of anti-Bcl-2 (1 : 5000; Abcam® Inc., Cambridge, England) and polyclonal antibodies of anti-caspase-3 (1 : 1000; Cell Signaling Technology Inc., Danvers, USA) and anti-*β*-actin (1 : 5000; Bioworld Technology Inc., Minnesota, USA), respectively. Then, the membranes were incubated with alkaline phosphatase goat anti-rabbit IgG (1 : 1000; ZSGB-Bio, Beijing, China; ZB-2308). Immunoreactive proteins were visualized by using BCIP/NBT kit (Invitrogen™, Camarillo, USA; 00-2209). Densitometric quantification was undertaken through using ImageJ Program.

### 2.10. Statistical Analysis

All the data were expressed as mean ± SD. Multiple comparisons between groups were made with one-way analysis of variance (ANOVA) using SPSS (17.0) software package. Values of *P* < 0.05 were considered statistically significant.

## 3. Results

### 3.1. Chemical Composition of NCBAE

**Figure 1:**
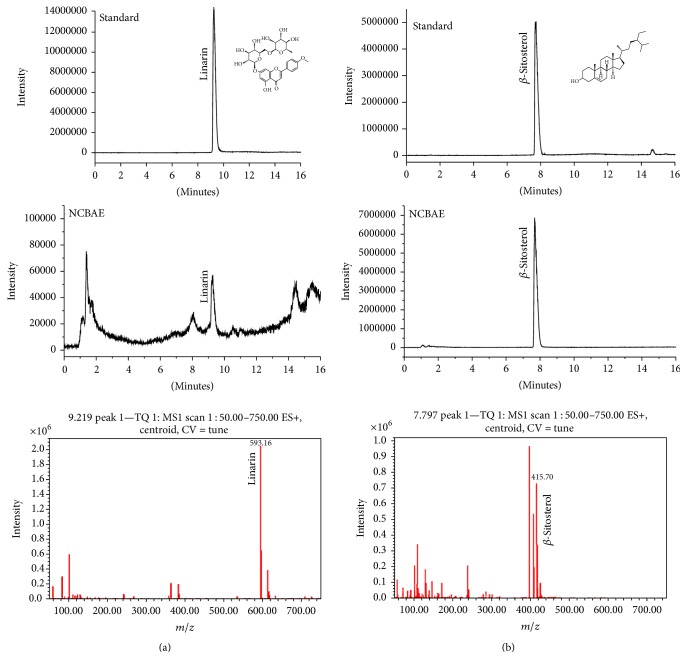
Identification of linarin (a) and *β*-sitosterol (b) in NCBAE by UPLC-MS method.

In this study, qualitative analysis of the chemical composition of NCBAE was performed by UPLC-MS. Experimental results showed that *m*/*z* ratios of linarin and *β*-sitosterol were 593.16 and 415.70, respectively. This data confirmed the existence of linarin and *β*-sitosterol in NCBAE (Figures 1(a) and 1(b)). HPLC results showed that the contents of linarin and *β*-sitosterol in NCBAE were 2.30% and 0.15%, respectively.

### 3.2. Comparison of SBP among Groups

**Figure 2:**
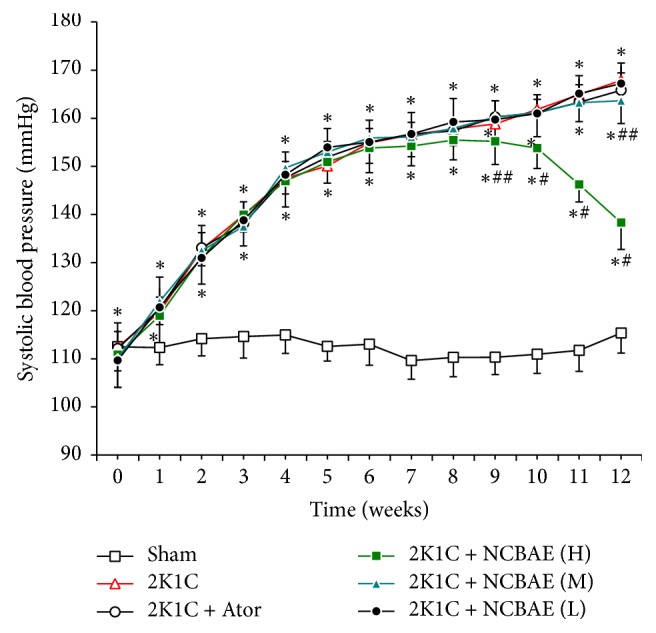
Development of SBP in the six experimental groups during a 12-week period. Thirteen time points of SBP were measured in each group. Data are expressed as the mean ± SD. *n* = 12. ^*∗*^*P* < 0.01 compared with sham-operated group. ^#^*P* < 0.01 compared with 2K1C group. ^##^*P* < 0.05 compared with 2K1C group.

Figure 2 indicates that SBP in the 2K1C-induced hypertensive model group was sharply higher than in the sham-operated group (*P* < 0.01). Treatment of NCBAE (H) and NCBAE (M) caused reduction in the development of SBP in 2K1C rats (*P* < 0.01, *P* < 0.05, resp., Figure 2). Comparison of the six groups at the end of the experiment showed that 2K1C group had SBP of 167.86 ± 3.04 mmHg, whereas rats treated with NCBAE (H) showed decreased SBP of 138.28 ± 5.56 mmHg.

### 3.3. Echocardiographic Parameters

**Figure 3:**
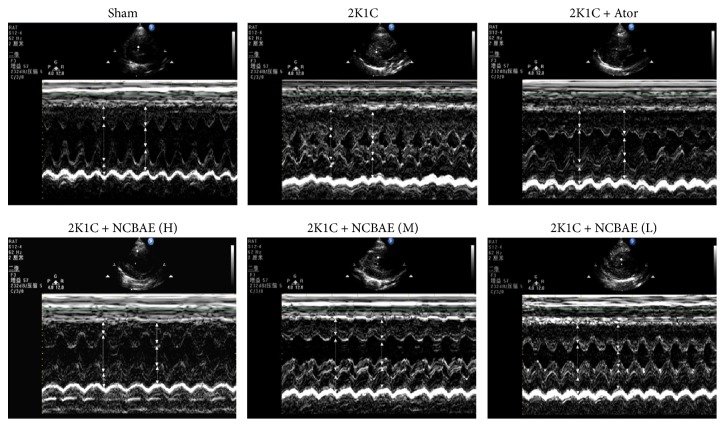
Representative echocardiography for each group.

Representative examples of each group are shown in Figure 3. As shown in the figure, a remarkably thickened left ventricular wall was observed in 2K1C hypertensive group compared with sham-operated group, while this change was alleviated after six-week NCBAE treatments. The echocardiographic parameters are shown in Table 1. Compared to the sham-operated group, the 2K1C group showed an increase in LVPWd, LVPWs, IVSd, and IVSs (*P* < 0.05, Table 1) and decreases in LVIDd, LVEF, and LVFS (*P* < 0.05, Table 1). NCBAE (H) treatment reduced LVPWd, LVPWs, IVSd, and IVSs (*P* < 0.05, Table 1) and enhanced LVEF (*P* < 0.05, Table 1) of 2K1C hypertensive rats. LVFS was relatively increased by treatment with NCBAE (H) compared to 2K1C group but did not show a significant difference (*P* > 0.05, Table 1). These data indicate that systolic dysfunction was evident in 2K1C hypertensive rat models, and the cardiac function was significantly improved in NCBAE (H) treatment group.

### 3.4. Body Weight, Left Ventricular Weight, and LVW/BW Ratio

As shown in Table 2, left ventricular weight (LVW) and LVW/BW ratio in the 2K1C group were significantly higher compared with the sham-operated group (*P* < 0.01). Compared with the 2K1C group, NCBAE (H) treatment and Ator treatment decreased LVW/BW ratio significantly.

### 3.5. Histopathological Study

**Figure 4:**
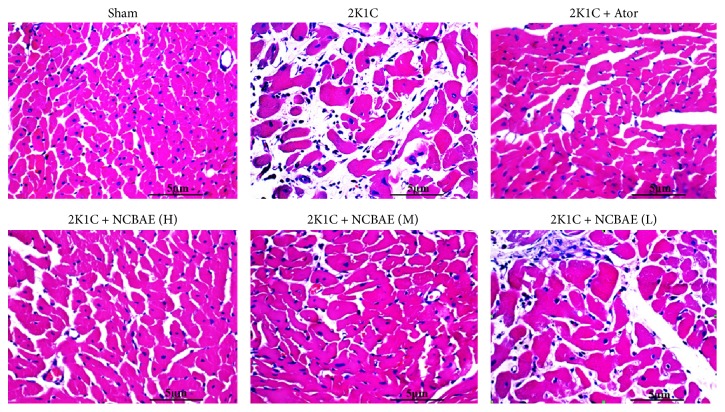
Effects of NCBAE on cardiac hypertrophy (representative pictures of myocardium stained with H&E, magnification: 400x).

**Figure 5:**
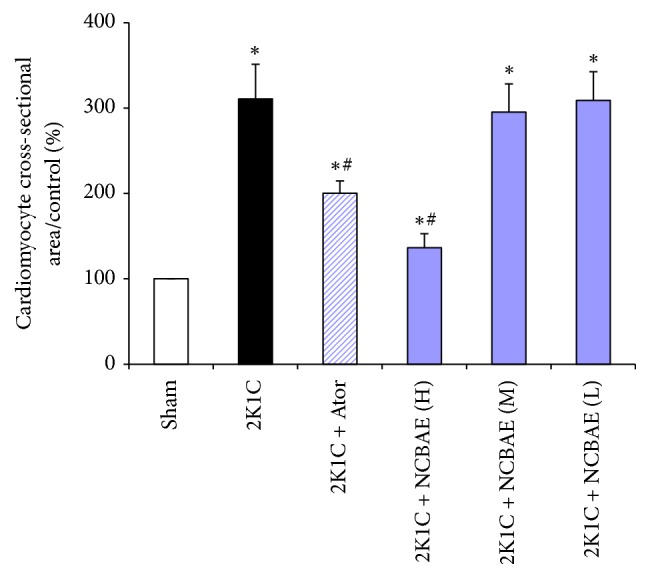
Quantitative analysis of cardiomyocyte CSA for samples of each group. Computer-based image analysis was applied for measurements. ^*∗*^*P* < 0.01 compared with sham-operated group. ^#^*P* < 0.01 compared with 2K1C group.

As shown in Figure 4, we found that the ventricular myocardium in sham-operated group showed normal architecture with normal interstitial space between cells, whereas in 2K1C rats, the abnormal architecture and increased interstitial space were observed. Moreover, cardiomyocyte CSA significantly increased in 2K1C rats compared with the sham-operated group (*P* < 0.01, Figures 4 and 5). These pathological changes were reduced significantly with NCBAE (H) treatment and Ator treatment (*P* < 0.01, Figures 4 and 5) compared with 2K1C group.

### 3.6. Serum NO and LDH

**Figure 6:**
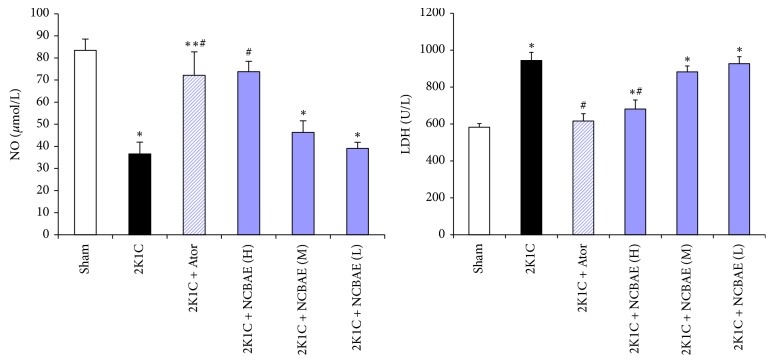
Inhibitory effects of treatment with NCBAE on 2K1C-induced reduction in serum NO and elevation of serum LDH in rats. ^*∗*^*P* < 0.01 compared with sham-operated group. ^#^*P* < 0.01 compared with 2K1C group.

As shown in Figure 6, 2K1C group showed a significant decrease in serum NO level and increase in serum LDH as compared to sham-operated group (*P* < 0.01). Treatment with NCBAE (H) and Ator for 6 weeks resulted in a significant increase in serum NO level compared with 2K1C group (*P* < 0.01, Figure 6). Treatment with NCBAE (H) and Ator also resulted in the reduction of serum LDH as compared to 2K1C group (*P* < 0.01, Figure 6). The rises in the activities of serum LDH and decreases in NO levels defined myocardial injury associated with 2K1C renovascular hypertension, and the treatment of NCBAE alleviated the injuries.

### 3.7. Western Blotting

**Figure 7:**
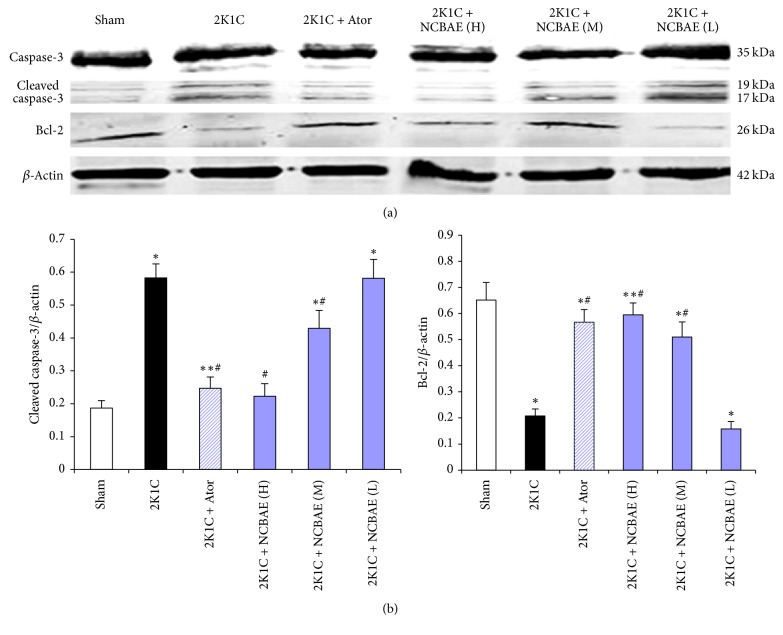
Effects of NCBAE on 2K1C-induced apoptosis. (a) The expression of Bcl-2 and cleaved caspase-3 protein was determined by western blotting. (b) Protein levels were measured by densitometry and presented as histograms. Bcl-2 and cleaved caspase-3 protein normalized to *β*-actin. ^*∗*^*P* < 0.01 compared with sham-operation group. ^*∗∗*^*P* < 0.05 compared with sham-operation group. ^#^*P* < 0.01 compared with 2K1C group.

Western blotting analysis showed that 2K1C renovascular hypertension led to cleavage of caspase-3 and downregulated expression of Bcl-2 in left ventricular tissue of rats (*P* < 0.01, Figure 7). Importantly, both the activation of caspase-3 and the downregulation of Bcl-2 observed in 2K1C hypertensive hearts were significantly attenuated by NCBAE (H), NCBAE (M), and Ator (*P* < 0.01, Figures 7(a) and 7(b)), suggesting that the modulation of apoptotic process by NCBAE might contribute to the beneficial effect on 2K1C hypertensive hearts.

## 4. Discussion

In recent years, a number of chemical compounds from NCB were obtained and their structures were identified as acaciin, ursolic acid, octacosanol, kanshone A, nardosinonediol, nardosinone, *β*-sitosterol, nardosinanone F, nardosinanone I, kanshone E, narchinol A, narchinol B, and others [15, 29]. In this research, we confirmed the existence of linarin and *β*-sitosterol in NCB aqueous extract, and these findings are consistent with the researches mentioned above. But there still are some other chemical components of the herbal extract that need to be further studied. A study demonstrates that nardosinanone F, nardosinanone I, nardosinonediol, kanshone E, narchinol A, and narchinol B protect rats' cardiomyocytes from H_2_O_2_ injury in vitro [15]. Ursolic acid has been shown to exhibit both antiapoptotic and antioxidative activities against endoplasmic reticulum stress-associated myocardial damage [30]. However, the effects of NCB on 2K1C hypertension-induced cardiac disorders have not been reported.

In our research, we used a recognized model of experimental hypertension, the 2K1C renovascular hypertensive rats [25, 31]. In these models, we found that SBP increased; LVPWd, LVPWs, IVSd, IVSs, LVW/BW ratio, and cardiomyocyte CSA increased; LVEF decreased; serum NO level decreased; and serum LDH increased as compared to sham-operated rats. These findings are consistent with previous reports on 2K1C hypertensive rat models [32–34]. Cardiomyocyte hypertrophy and cardiac remodeling caused by hypertension led to left ventricular dysfunction [34]. The left ventricular parameters and morphological changes of these models, together with their decreased serum NO level and increased cardiac injury marker LDH, showed the damaged status of hypertensive hearts. These findings indicate that the 2K1C procedure induced hypertension and left ventricular dysfunction after 6 weeks of renal artery clipping.

During six-week NCBAE treatments, we observed that the extract showed an antihypertensive effect, decreased SBP of hypertensive rats compared with 2K1C group. We also found that NCBAE reduced LVPWd, LVPWs, IVSd, IVSs, LVW/BW ratio, and cardiomyocyte CSA and increased LVEF of hypertensive rats compared with 2K1C group. These results indicate that NCBAE regulates BP and attenuates ventricular hypertrophic remodeling in 2K1C hypertensive rats. NO, as an endothelium-derived relaxing factor [35, 36], plays an important role in the regulation of the cardiovascular system [37, 38]. Studies have found that reduced NO production caused by endothelial dysfunction is an important factor in the development of cardiovascular diseases such as hypertension [39], and left ventricular dysfunction is closely associated with reduced NO production [22]. Besides, hypertension causes myocardial damage resulting in enhanced cell membrane permeability or even rupture that leads to leakage of cardiac enzyme LDH. Serum LDH is one of the important enzymes for the evaluation of cardiac injury and heart failure [40] and is often used as a marker for tissue breakdown [41]. In this study, a six-week treatment with NCBAE inhibited 2K1C-induced reduction in serum NO and elevation of serum LDH. These results indicate that NCBAE has a protective effect on 2K1C hypertensive hearts.

Cardiac dysfunction is also associated with myocardial apoptosis [42, 43]. Apoptosis is characterized by the morphological features including shrinkage of the cell, condensation of the cytoplasm and chromatin, “blebbing” of the cell membrane, and fragmentation of the nucleus [18]. The intrinsic pathway that involves disruption of mitochondrial membrane integrity and the extrinsic pathway activated by proapoptotic receptor signals at cellular surface are the two major apoptotic signal pathways; both of them activate caspases family and lead to the cleavage of multiple intracellular substrates [44]. The activation of caspase-3 results in apoptosis, and this process is regulated by antiapoptotic protein Bcl-2 [45]. To gain more insight into the mechanism of the NCBAE function, we examined the cardiac protein expression of Bcl-2 and caspase-3. In our study, 2K1C renovascular hypertension results in cardiac hypertrophic response, which is characterized by LVW/BW ratio and cardiomyocyte CSA increase compared with sham-operated rats. However, the results from western blotting analysis showed that expression of Bcl-2 decreased, and cleaved caspase-3 level increased obviously. These results suggested that the formation process of cardiac hypertrophy may be along with myocardial apoptosis. And during this process, the remaining myocardial cells were compensatory excessive contracted in order to prevent cardiac dysfunction caused by reduced cardiomyocytes. Interestingly, our study showed that NCBAE intervention upregulated the expression of Bcl-2 but reduced cleaved caspase-3 levels dose-dependently compared with 2K1C hypertensive rats. These observations suggest that NCBAE might protect hypertensive hearts through regulating Bcl-2 and caspase-3 against apoptosis, resulting ultimately in improving cardiac function.

In conclusion, increased BP, reduced NO level, increased activity of LDH, and imbalance of Bcl-2 and caspase-3 increase myocardial hypertrophy, which exacerbates cardiac dysfunction. This cardiac injury and pathological remodeling can be reversed to some degree by NCBAE treatment. As a traditional herbal medicine, NCBAE may become a potent therapy for hypertensive cardiac disorders. For the deeper recognition, further studies are required to elucidate specific mechanisms of NCBAE intervention on 2K1C hypertension-induced cardiac injury, and the relevant effective chemical compounds of the herbal extract need to be further explored as well.

## Figures and Tables

**Table 1:** The echocardiographic parameters in each group.

Group	Sham (*n* = 12)	2K1C (*n* = 12)	2K1C + Ator (*n* = 12)	2K1C + NCBAE (H) (*n* = 12)	2K1C + NCBAE (M) (*n* = 12)	2K1C + NCBAE (L) (*n* = 12)
LVPWd (mm)	1.55 ± 0.24	1.72 ± 0.28^*∗*^	1.68 ± 0.10	1.57 ± 0.14^#^	1.76 ± 0.16^*∗*^	1.74 ± 0.14^*∗*^
LVPWs (mm)	2.52 ± 0.31	2.78 ± 0.16^*∗*^	2.68 ± 0.10^*∗*^	2.55 ± 0.18^#^	2.82 ± 0.10^*∗*^	2.86 ± 0.10^*∗*^
LVIDd (mm)	6.62 ± 0.52	5.83 ± 0.39^*∗*^	5.81 ± 0.68^*∗*^	5.97 ± 0.58^*∗*^	5.87 ± 0.56^*∗*^	5.82 ± 0.51^*∗*^
LVIDs (mm)	4.03 ± 0.44	3.85 ± 0.48	3.82 ± 0.40	3.86 ± 0.47	3.83 ± 0.34	3.82 ± 0.43
IVSd (mm)	1.44 ± 0.23	1.89 ± 0.28^*∗*^	1.52 ± 0.11^#^	1.56 ± 0.09^#^	1.74 ± 0.16^*∗*#^	1.75 ± 0.08^*∗*^
IVSs (mm)	2.51 ± 0.31	2.92 ± 0.29^*∗*^	2.83 ± 0.32^*∗*^	2.63 ± 0.20^#^	2.89 ± 0.25^*∗*^	2.84 ± 0.28^*∗*^
LVEF (%)	70.20 ± 5.05	59.71 ± 7.65^*∗*^	62.65 ± 4.38^*∗*^	68.74 ± 7.63^#^	59.99 ± 6.88^*∗*^	58.68 ± 8.05^*∗*^
LVFS (%)	39.17 ± 3.73	33.89 ± 6.81^*∗*^	33.98 ± 5.22^*∗*^	35.19 ± 5.56	34.37 ± 6.27^*∗*^	34.31 ± 4.62^*∗*^

LVPWd: left ventricular posterior wall at end diastole; LVPWs: left ventricular posterior wall at end systole; LVIDd: left ventricular internal diameter at end diastole; LVIDs: left ventricular internal diameter at end systole; IVSd: interventricular septal diameter at end diastole; IVSs: interventricular septal diameter at end systole; LVEF: left ventricular ejection fraction; LVFS: left ventricular fractional shortening. All values are mean ± SD. ^*∗*^*P* < 0.05 compared with sham-operation group. ^#^*P* < 0.05 compared with 2K1C group.

**Table 2:** BW, LVW, and LVW/BW in each group.

Group	LVW (mg)	BW (g)	LVW/BW (mg/g)
Sham (*n* = 12)	750.00 ± 26.29	366.25 ± 13.96	2.05 ± 0.08
2K1C (*n* = 12)	946.67 ± 32.00^*∗*^	365.58 ± 4.87	2.59 ± 0.09^*∗*^
2K1C + Ator (*n* = 12)	918.33 ± 28.55^*∗*##^	368.92 ± 4.17	2.49 ± 0.06^*∗*#^
2K1C + NCBAE (H) (*n* = 12)	898.33 ± 41.30^*∗*#^	365.33 ± 8.75	2.46 ± 0.11^*∗*#^
2K1C + NCBAE (M) (*n* = 12)	943.33 ± 20.60^*∗*^	370.42 ± 9.52	2.55 ± 0.10^*∗*^
2K1C + NCBAE (L) (*n* = 12)	944.17 ± 31.47^*∗*^	365.75 ± 7.39	2.58 ± 0.10^*∗*^

LVW: left ventricular weight; BW: body weight; LVW/BW: left ventricular weight to body weight ratio. All values are mean ± SD. ^*∗*^*P* < 0.01 compared with sham-operation group. ^#^*P* < 0.01 compared with 2K1C group. ^##^*P* < 0.05 compared with 2K1C group.

## Abbreviations

NCB: * Nardostachys chinensis* Batalin
NCBAE: Aqueous extract of* Nardostachys chinensis* Batalin
Ator: Atorvastatin
2K1C: Two-kidney one-clip
Ang II: Angiotensin II
BP: Blood pressure
SBP: Systolic blood pressure
BW: Body weight
LVW: Left ventricular weight
LVW/BW: Left ventricular weight/body weight
CSA: Cross-sectional area
LVPWd: Left ventricular posterior wall at end diastole
LVPWs: Left ventricular posterior wall at end systole
LVIDd: Left ventricular internal diameter at end diastole
LVIDs: Left ventricular internal diameter at end systole
IVSd: Interventricular septal diameter at end diastole
IVSs: Interventricular septal diameter at end systole
LVEF: Left ventricular ejection fraction
LVFS: Left ventricular fractional shortening
NO: Nitric oxide
LDH: Lactate dehydrogenase
i.p.: Intraperitoneal.
